# Personality traits and digital challenges in Honduran adults: exploring the Dark and Light Triads’ influence on internet gaming disorder and technology-related conflicts

**DOI:** 10.3389/fpubh.2025.1485264

**Published:** 2025-02-19

**Authors:** Claudio J. Mejía-Suazo, Miguel Landa-Blanco, Gliver Aarón Mejía-Suazo, Carlos A. Martínez-Martínez

**Affiliations:** ^1^School of Biology, Faculty of Sciences, National Autonomous University of Honduras, Tegucigalpa, Honduras; ^2^School of Psychological Sciences, Faculty of Social Sciences, National Autonomous University of Honduras, Tegucigalpa, Honduras; ^3^Faculty of Medical Sciences, National Autonomous University of Honduras, Tegucigalpa, Honduras

**Keywords:** Dark Triad, Light Triad, behavioral addiction, gaming disorder, mobile phone conflicts, problematic internet use

## Abstract

**Introduction:**

The present study analyzed the effects of Light and Dark Triad traits scores on Internet Gaming Disorder, intrapersonal and interpersonal conflicts related to internet consumption, conflicts, and communicational emotional usage related to mobile phones. Light Triad traits include Faith in Humanity, Humanism, and Kantianism. Dark Triad traits include Machiavellianism, Narcissism, and Psychopathy.

**Methods:**

The sample consisted of 450 adults of the Honduran population, of which 55.33% were women, and 44.67% were men, with an average mean age of 25.52 years (*SD* = 6.79).

**Results:**

Results indicate that Machiavellianism and Psychopathy scores have significant effects over Internet Gaming Disorder scores. Intrapersonal conflicts related to internet consumption were negatively affected by Narcissism scores and positively affected by Machiavellianism. Psychopathy scores explained interpersonal conflicts related to internet consumption scores. On the other hand, conflicts related to mobile phone usage were negatively affected by Narcissism and Kantianism, while Machiavellianism had positive effects. Finally, communicational emotional usage related to mobile phones was significantly affected by Machiavellianism.

**Discussion:**

When comparing by respondent’s sex, Machiavellianism consistently emerges as a key predictor; however, its effects tend to be stronger in men. In contrast, Psychopathy, Kantianism, and Narcissism exhibit more sex-specific associations, influencing females and males differently across digital behaviors and conflicts.

## 1 Introduction

### 1.1 Dark Triad and Light Triad of personality

In recent years, the scientific community has given increased attention to the study of personality traits in adult populations. For instance, there is a particular interest in understanding the characteristics of people who successfully occupy essential societal roles regardless of exhibiting a psychopathic profile (1, 2). This phenomenon suggests that specific individuals with psychopathic traits may develop mechanisms to regulate their behavior and integrate effectively into society (3).

Paulhus and Williams (4) initially coined the term Dark Triad (DT) to describe a unique combination of personality traits, specifically Machiavellianism, Narcissism, and Psychopathy. Socially offensive behaviors characterize these traits but do not reach clinical or forensic levels, classified as subclinical. Machiavellianism involves manipulation to exploit others, Narcissism includes fantasies of power and grandiosity, and Psychopathy is marked by a lack of empathy and remorse, along with impulsive behaviors in search of excitement (5–7).

While the Dark Triad traits are linked to socially offensive behaviors, they exhibit a complexity beyond mere maladaptive tendencies. Recent research has also highlighted that these traits can serve adaptive functions in specific contexts, indicating that their effects may vary depending on the situation (5, 8).

On the other hand, the Light Triad (LT) emerges as a complementary measure to explore positive aspects of personality, such as Kantianism, Humanism, and Faith in Humanity (9, 10). While the Dark Triad focuses on traits associated with self-interest and manipulation, the Light Triad highlights attributes that foster connection and moral integrity (11). Kantianism reflects the ethical principle of treating others as ends in themselves rather than as means to an end (12). Humanism underscores every individual’s inherent value and dignity, promoting a compassionate and respectful outlook (13). Faith in Humanity is grounded in an optimistic belief in the fundamental goodness of people (14). Together, these traits provide a framework for understanding the constructive and prosocial dimensions of personality, counterbalancing the more antagonistic traits of the Dark Triad.

These personality traits can be modulated by the environment and individual experiences, generating positive and negative emotions and reactions (2, 14). In the context of technological evolution, behavioral addictions have gained relevance, particularly concerning excessive use of mobile phones, video games, and the internet (15, 16). Research suggests that individuals with higher levels of Dark Triad traits—specifically Machiavellianism and Narcissism—may be more prone to engage in these behavioral addictions as they seek validation and power through digital platforms (17). In contrast, those exhibiting Light Triad traits, such as Humanism and Faith in Humanity, might leverage technology to enhance social connections and foster well-being (18). This duality underscores the complex interplay between personality traits and behavioral addictions, highlighting how these traits can influence susceptibility to maladaptive behaviors in a rapidly evolving digital landscape.

### 1.2 Intrapersonal and interpersonal conflicts related to problematic internet use

Problematic internet use is an excessive and uncontrollable urge to access the Internet, leading to significant negative consequences in the individual’s personal, social, and occupational life (19, 20). This excessive connectivity can trigger intrapersonal conflicts, which manifest as internal struggles characterized by anxiety, low self-esteem, and self-regulation problems. Furthermore, these intrapersonal challenges contribute to interpersonal conflicts, affecting social relationships and generating isolation, dependence on social networks, and family tensions (21, 22). Researchers are becoming increasingly interested in this topic given the increase in the dependency that humans have on the internet (23, 24).

Problematic internet use is characterized by a strong urge to stay permanently connected to the internet, paired with a feeling of anxiety and anguish when disconnected. One explanation for excessive and uncontrolled internet use is that people with those behaviors must escape their problems and reality (24, 25). In addition, problematic internet usage is associated with physical and psychological issues such as loneliness, low self-esteem, social isolation, lack of sleep, fatigue, anxiety, and symptoms of depression, which can be detrimental in personal, academic, and professional aspects of life (26, 27).

Problematic internet use is increasingly recognized as a source of intrapersonal and interpersonal conflicts, each with distinct but interconnected consequences. Intrapersonal conflicts pertain to the internal psychological effects of internet use, focusing on how it shapes emotional well-being, time perception, and overall life satisfaction. Individuals may rely on the internet as a coping mechanism to escape from real-life challenges, develop a sense of dependence, and experience feelings of emptiness when disconnected from it. This dimension also encompasses issues such as distractibility, the loss of time while browsing, and a preference for online interactions over face-to-face communication (28).

On the other hand, interpersonal conflicts address the broader social and behavioral repercussions of excessive internet use, particularly its impact on relationships, daily responsibilities, and productivity. This includes the potential for internet addiction, withdrawal symptoms when disconnected, and the negative influence of internet use on academic or professional performance (28, 29). Together, these two dimensions offer a comprehensive understanding of the multifaceted consequences of problematic internet use, spanning from individual psychological struggles to broader social and behavioral disruptions. However, while uncontrolled internet use can have adverse effects, responsible use has been observed to enhance personal, academic, and professional success. Additionally, it facilitates cross-cultural interaction, access to information, and economic development (30).

### 1.3 Communicational emotional usage related to mobile phones

Mobile phones have experienced significant advancements over the past few decades. Initially limited to essential communication functions such as voice calls and text messaging, they have since evolved into sophisticated multifunctional devices. Contemporary mobile phones now serve as central tools for various applications, including photography, gaming, music streaming, voice recording, and numerous other purposes (31). Mobile phones are also connected to the internet, allowing interactions with people worldwide through social media platforms like Instagram, Facebook, WhatsApp, and X. Consequently, mobile phones have become one of the most used technologies for information and communication worldwide (32, 33).

The multiple benefits of mobile phones are evident and have become a fundamental element of daily human life. However, the disproportionate use of mobile phones can have dangerous effects. Excessive mobile phone use can cause the reduction of physical activity, sleep disturbances, depression, psychological morbidity, and ultimately develop into dependency and addictive use, producing side effects similar to those in people who abuse psychostimulants (loss of control, desire, abstinence, and relapse). Evidence has shown that conflicts related to the use of mobile phones can cause depression, loss, and isolation when experiencing withdrawal, leading to low performance both in personal and professional aspects (31–33). These findings have led the World Health Organization (WHO) to consider the excessive use of mobile phones as a public health concern (34). In the Honduran context, recent studies have found that increased social media consumption is linked to higher levels of suicidal ideation, depression, and anxiety, along with a decrease in self-esteem among young adults (35–37).

### 1.4 Internet Gaming Disorder

Gaming Disorder refers to recurrent dysfunctional behavioral patterns related to excessive video game consumption, which results in a significant deterioration in personal, social, and occupational well-being (38). Personality traits most strongly linked to compulsive gaming behavior include neuroticism, aggression, hostility, and sensation-seeking (39). Gaming Disorder has also been compared to substance use disorders since the brain reacts similarly when playing a competition-like video game and when using psychostimulant drugs (40). Specifically, Internet Gaming Disorder (IGD) has been identified in a significant proportion of the population (41), particularly among male and young users (42).

The IGD can lead to several negative psychological consequences: neglecting responsibilities, reduced quality time with loved ones, and sleep alterations. In extreme cases, these individuals may present physical problems such as epileptic seizures, auditory hallucinations, and tenosynovitis. Therefore, the study and intervention of IGD have become increasingly important among the general population (43). However, not everything is negative regarding video games. Studies have shown that playing violent video games can be associated with increased visuospatial cognition (44), improved mood, and essential prosocial skills when the games have cooperative or supportive goals (45). They also have potential value in improving self-esteem, supporting psychotherapeutic treatment, and conflict resolution (46).

The Interaction of Person-Affect-Cognition-Execution (I-PACE) model is a theoretical framework to explain the development and persistence of behavioral addictions, such as IGD and problematic technology use. It emphasizes the interaction between individual predispositions (e.g., personality traits and environmental influences), affective and cognitive processes (e.g., emotional regulation and cognitive distortions), and executive functioning (e.g., impulse control and decision-making). These interactions reinforce maladaptive behaviors over time, often driven by the pursuit of emotional relief or perceived rewards (47).

The I-PACE model is highly relevant to this research as it provides a structured approach to understanding how personality traits, particularly the Light and Dark Triad traits, contribute to problematic digital behaviors and associated interpersonal and intrapersonal conflicts. By focusing on the interaction of personal characteristics with emotional and cognitive processes, the model helps explain individual differences in susceptibility to behaviors like IGD and mobile phone-related challenges. Additionally, it offers a framework for analyzing how these patterns vary across demographic factors, such as gender, thereby supporting a deeper investigation into the mechanisms underlying digital addiction and conflict.

### 1.5 Purpose of the study

**Figure 1:**
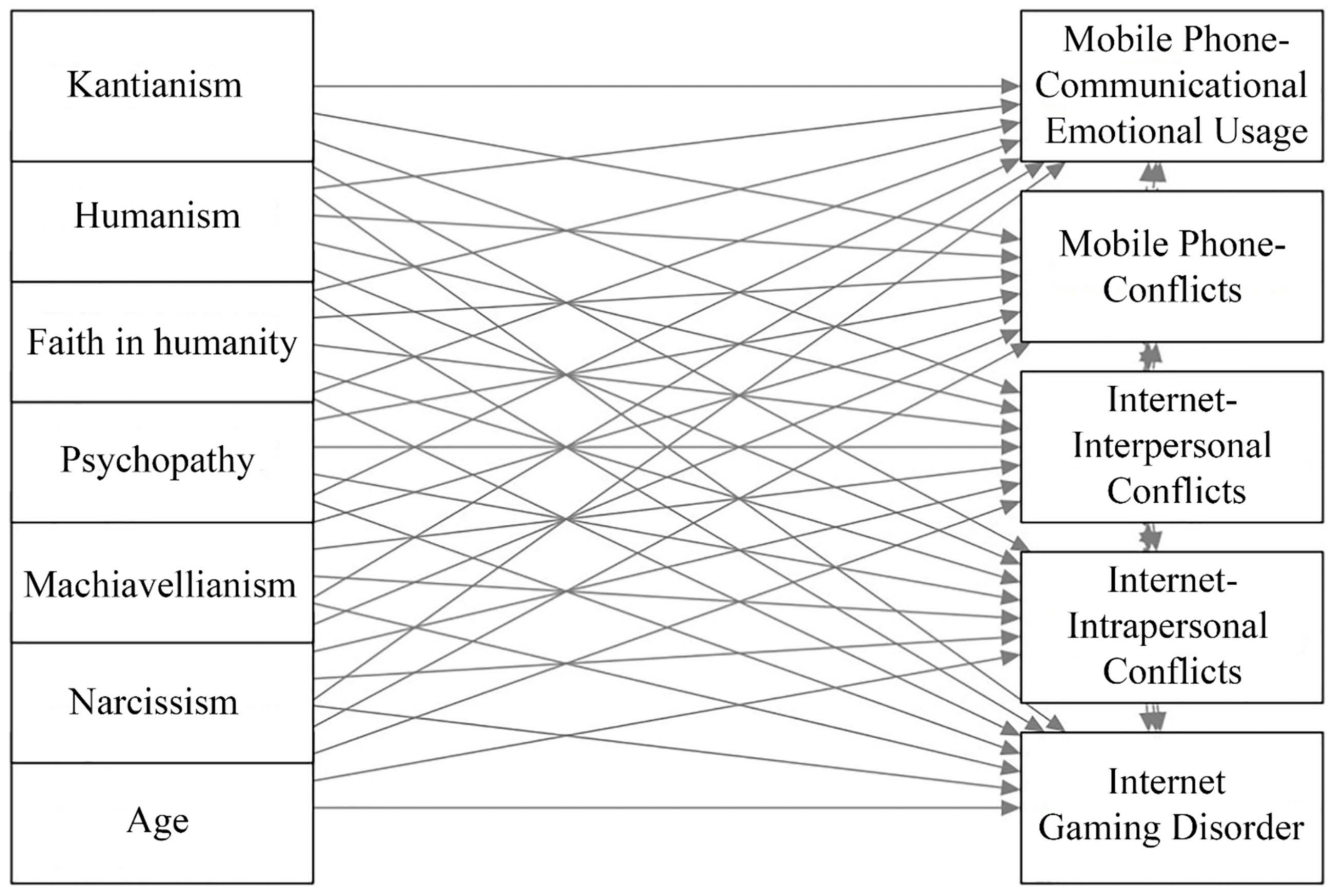
Path analysis model. Representation of the relationships between personality traits (Dark and Light Triads) and age with Internet Gaming Disorder, internet-related interpersonal and intrapersonal conflicts, and mobile phone communicational-emotional usage and conflicts.

Considering all the above, the present study examines the effects of age, sex, Dark Triad and Light Triad personality traits on IGD, intrapersonal and interpersonal conflicts associated with internet use, and conflicts and communicational-emotional usage related to mobile phones in a sample of Honduran adults (see Figure 1). A secondary objective is to explore sex differences in the relationships among these variables. Thus, the following hypotheses are stated:

*Hypothesis 1*: Dark Triad and Light Triad personality traits, along with age and sex, will significantly predict Internet Gaming Disorder, intrapersonal and interpersonal conflicts associated with internet use, and conflicts and communicational-emotional usage related to mobile phones in a sample of Honduran adults.

*Hypothesis 2*: There will be significant differences in the relationships between Dark Triad and Light Triad personality traits, age, and the outcomes (Internet Gaming Disorder, internet-related conflicts, and mobile phone use) between men and women, indicating different dynamics in these variables across sexes.

This manuscript is crucial for public health as it sheds light on the complex relationships between personality traits—specifically the Light and Dark Triads—and problematic digital behaviors, including IGD and conflicts arising from internet and mobile phone use. Understanding how traits like Machiavellianism, Narcissism, and Psychopathy influence these behaviors is vital for developing effective public health interventions aimed at reducing the psychological and social harms associated with excessive digital consumption. By focusing on the Honduran population, the study contributes to the global understanding of these issues and addresses a gap in research within this cultural context, often underrepresented in public health studies.

## 2 Materials and methods

### 2.1 Data collection techniques

#### 2.1.1 Questionnaire of Experiences Related to the Internet (QERI)

The Questionnaire of Experiences Related to the Internet (QERI) consists of 10 items distributed in two factors: Intrapersonal Conflicts (*α* = 0.74) and Interpersonal Conflicts (*α* = 0.75) (29). The scale uses a Likert-type response set of 4 points (1 = “never,” 2 = “almost never,” 3 = “almost always,” 4 = “always”); higher mean scores indicate a higher intensity of internet-related conflicts.

#### 2.1.2 Questionnaire of Experiences Related to the Mobile Phone (QERMP)

The Questionnaire of Experiences Related to the Mobile Phone (QERMP) consists of 10 items distributed in two factors: Conflicts (*α* = 0.81) and Communicational/Emotional Use (*α* = 0.75) (29). The scale uses a Likert-type response set of 4 points (1 = “never,” 2 = “almost never,” 3 = “almost always,” 4 = “always”); higher mean scores indicate a higher intensity of mobile phone experiences.

#### 2.1.3 Internet Gaming Disorder Scale-Short Form (IGDS9-SF)

The Internet Gaming Disorder Scale-Short Form (IGDS9-SF) consists of nine items based upon the Diagnostic and Statistical Manual of Mental Disorders fifth edition (DSM-5) criteria for Internet Gaming Disorder. The IGDS9-SF uses a five-point Likert-type response set (1 = “never,” 2 = “rarely,” 3 = “sometimes,” 4 = “often,” 5 = “very often”). The scale possesses adequate reliability (*α* = 0.85) and validity (48). Higher mean scores indicate a higher intensity of Internet Gaming Disorder symptoms.

#### 2.1.4 Short Dark Triad (SD3)

The Short Dark Triad (SD-3) measures Machiavellianism (*α* = 0.76), Narcissism (*α* = 0.78), and Psychopathic traits (*α* = 0.73). The SD-3 consists of 27 items, with a five-point Likert-type response set (1 = “disagree strongly,” 2 = “disagree,” 3 = “neither agree nor disagree,” 4 = “agree,” 5 = “agree strongly”). The SD-3 has also been proven to be a valid measure of Dark Triad traits (49). A higher mean score indicates a higher trait intensity.

#### 2.1.5 Light Triad Scale (LTS)

The Light Triad Scale (LTS) is a 12-item questionnaire that measures Faith in Humanity (*α* = 0.80), Humanism (*α* = 0.76), and Kantianism (*α* = 0.67), with a total internal consistency of 0.84, as measured through Cronbach’s alpha. The responses use a Likert-type format with 5 points (1 = disagree strongly, 2 = disagree, 3 = neither agree nor disagree, 4 = agree, 5 = agree strongly). The LTS is considered a valid measurement (14), that has already been used in the Honduran population (35–37). A higher mean score indicates a higher trait intensity.

#### 2.1.6 Demographic Questionnaire

The Demographic Questionnaire collected data regarding the respondent’s sex (male = 0; female = 1), age, country of residence, a self-reported measure of video consumption (hours per day), and mobile phone usage (hours per day).

### 2.2 Sample

The sample consisted of 450 respondents selected through a non-probabilistic approach. Participants were recruited through the online distribution of questionnaires via social media platforms such as Facebook, Instagram, and X. The recruitment process employed a snowball sampling technique, starting with a few initial participants who were encouraged to share the survey link with their personal networks. These participants then passed the survey along to others in their social circles, further expanding the pool of respondents. The inclusion criteria were: (1) being 18 years or older, (2) currently living in Honduras, and (3) agreeing to the informed consent. The sample included 259 women (55.33%) and 201 men (44.67%); the respondent’s mean age was 25.52 years (*SD* = 6.79; minimum age = 18; maximum age = 56). On average, the sample reported playing video games 5.03 h a day (*SD* = 4.34) and using the mobile phone for 8.25 h a day (*SD* = 4.69).

### 2.3 Ethical considerations

Informed consent was included at the beginning of each questionnaire. It stated the purpose of the study, a confidentiality statement, and the researcher’s contact information.

### 2.4 Data analyses

First, total mean scores and standard deviations were determined for each subscale included in the study. Statistical analyses were made using Jamovi 2.3.28 (50) regression and pathj modules. An internal consistency analysis was also made using Cronbach’s alpha. Then, simultaneous input linear regression models were used to determine the relationship between variables. Specifically, outcome variables included: Internet Gaming Disorder scores, Intrapersonal and Interpersonal conflicts related to internet consumption, Communicational Emotional Usage, and Conflicts related to mobile phone usage. Predictor variables included: age, sex, Narcissism, Machiavellianism, Psychopathy, Humanism, Kantianism, and Faith in Humanity. Variance Inflation Factor (VIF), determination coefficients (*R^2^*), post-hoc power, and effect sizes (*f^2^*) were calculated for each model. Effect sizes were classified according to Cohen’s recommendations: *f^2^* = 0.02 is classified as small, *f^2^* = 0.15 as a medium, and *f^2^* = 0.35 as large (51). Subsequently, a multigroup path analysis was conducted based on the respondent’s sex to evaluate differences between males and females. While the regression models provide initial insights, readers should prioritize the path analysis results, which account for correlations among outcomes and mitigate Type I error risks. All significance was tested at a 95% confidence level.

## 3 Results

The general description of Dark Triad subscales indicates that Narcissism traits have the highest scores (*M* = 2.90; *SD* = 0.60), followed by Machiavellianism (*M* = 2.74; *SD* = 0.71) and Psychopathy (*M* = 2.01; *SD* = 0.74). On the other hand, Humanism scores (*M* = 3.42; *SD* = 0.81) were the highest among the Light Triad traits, followed by Kantianism (*M* = 3.87; *SD* = 0.74) and Faith in Humanity (*M* = 3.42; *SD* = 0.81).

The average Internet Gaming Disorder Scale-Short Form score was 1.65 (*SD* = 0.81). In the Questionnaire of Experiences Related to the Internet (*M* = 2.13; *SD* = 0.49), Intrapersonal Conflicts (*M* = 2.35; *SD* = 0.58) were more prevalent than interpersonal conflicts (*M* = 1.91; *SD* = 0.55). Meanwhile, in the Questionnaire of Experiences Related to Mobile Phone (*M* = 1.95; *SD* = 0.50), the Communicational Emotional Usage subscale had higher mean scores (*M* = 2.16; *SD* = 0.54) than the Conflicts subscale (*M* = 1.69; *SD* = 0.59). The correlation between variables is presented in Table 1.

**Table 1:** Correlation between variables included in the study.

Variable	1	2	3	4	5	6	7	8	9	10	11	12	13
1. Narcissism	—												
2. Machiavellianism	0.23 ***	—											
3. Psychopathy	0.222 ***	0.594 ***	—										
4. Humanism	0.011	−0.264 ***	−0.258 ***	—									
5. Faith in humanity	0.042	−0.214 ***	−0.236 ***	0.427 ***	—								
6. Kantianism	−0.118 *	−0.174 ***	−0.205 ***	0.303 ***	0.179 ***	—							
7. Internet Gaming Disorder	0.103 *	0.392 ***	0.443 ***	−0.146 **	−0.096 *	−0.161 ***	—						
8. Internet-Intrapersonal Conflicts	0.003	0.34 ***	0.239 ***	−0.093 *	−0.031	−0.11 *	0.355 ***	—					
9. Internet-Interpersonal Conflicts	0.015	0.203 ***	0.221 ***	−0.113 *	−0.033	−0.092	0.326 ***	0.523 ***	—				
10. Internet-Total	0.009	0.313 ***	0.263 ***	−0.116 *	−0.036	−0.115 *	0.39 ***	0.881 ***	0.864 ***	—			
11. Mobile Phone-Conflicts	−0.066	0.239 ***	0.212 ***	−0.119 *	−0.076	−0.179 ***	0.35 ***	0.481 ***	0.648 ***	0.643 ***	—		
12. Mobile Phone-Communicational Emotional Usage	0.073	0.299 ***	0.21 ***	−0.032	−0.085	−0.066	0.326 ***	0.648 ***	0.52 ***	0.672 ***	0.546 ***	—	
13. Mobile Phone-Total	0.001	0.304 ***	0.24 ***	−0.088	−0.091	−0.142 **	0.385 ***	0.637 ***	0.668 ***	0.747 ***	0.891 ***	0.866 ***	—
14. Age	0.008	−0.112 *	−0.107 *	−0.032	0.068	0.054	−0.115 *	−0.159 ***	−0.183 ***	−0.196 ***	−0.221 ***	−0.205 ***	−0.243 ***

Correlation coefficients were calculated with Pearson’s *r*.

*p < 0.05, **p < 0.01, ***p < 0.001.

Regression models were used to determine the influence of sex, age, and Light and Dark Triad traits over the scores of Internet Gaming Disorder, intrapersonal and interpersonal conflicts related to internet consumption, conflicts, and communicational emotional usage related to mobile phones. In this sense, the model significantly explains Internet Gaming Disorder scores (*R^2^* = 0.26), with Age and being female having negative effects, while Machiavellianism and Psychopathy scores have significant positive effects. The model achieved a large effect size, *f^2^* = 0.356.

The model significantly explained intrapersonal conflicts related to internet consumption, with a medium effect size (*R^2^* = 0.15; *f^2^* = 0.173). Narcissism scores negatively affect intrapersonal conflicts, while Machiavellianism scores are positively associated with such outcomes. On the other hand, the model accounted for 9% of interpersonal conflicts related to internet consumption scores (*R^2^* = 0.090; *f^2^* = 0.100); this effect size is classified as small. Age has significant negative effects, and Psychopathy is positively related to interpersonal conflicts.

The model also significantly explained conflicts related to mobile phone usage (*r^2^* = 0.14; *f^2^* = 0.158); the achieved effect size is classified as medium. Narcissism, Kantianism, and age are inversely related to such conflicts, while Machiavellianism has positive effects. Finally, the proposed model significantly predicts communicational emotional usage related to mobile phones (*R^2^* = 0.14; *f^2^* = 0.157). Age is a significant negative predictor, while being female and Machiavellianism are positively related to communicational emotional usage. The achieved effect size is classified as medium (see Table 2).

**Table 2:** Regression models explaining Internet Gaming Disorder, Internet-related Conflicts and Mobile Phone-related Conflicts.

Outcome	Predictor	*β*	SE	Standardized	*p*	95% CI		*r^2^*	*f^2^*	*F*
						*LL*	*UL*			
Internet Gaming Disorder	(Intercept)	1.111	0.381		0.004	0.363	1.86	0.26	0.356 (>0.99)	19.697 (*p* < 0.001)
	Age	−0.01	0.005	−0.083	**0.049**	−0.02	−0.01			
	Sex	−0.323	0.073	−0.199	**<0.001**	−0.467	−0.18			
	Narcissism	−0.021	0.058	−0.016	0.716	−0.135	0.093			
	Machiavellianism	0.191	0.06	0.167	**0.002**	0.074	0.309			
	Psychopathy	0.292	0.059	0.266	**<0.001**	0.177	0.408			
	Faith in humanity	0.029	0.046	0.029	0.532	−0.061	0.119			
	Humanism	−0.002	0.056	−0.001	0.978	−0.111	0.108			
	Kantianism	−0.045	0.048	−0.041	0.352	−0.14	0.05			
Internet-Intrapersonal Conflicts	(Intercept)	2.025	0.294		<0.001	1.447	2.602	0.15	0.173 (>0.99)	9.564 (*p* < 0.001)
	Age	−0.01	0.004	−0.112	**0.013**	−0.017	−0.002			
	Sex	0.063	0.056	0.054	0.264	−0.048	0.174			
	Narcissism	−0.091	0.045	−0.094	**0.042**	−0.179	−0.003			
	Machiavellianism	0.263	0.046	0.321	**<0.001**	0.172	0.354			
	Psychopathy	0.062	0.045	0.078	0.175	−0.028	0.151			
	Faith in humanity	0.059	0.035	0.083	0.095	−0.01	0.129			
	Humanism	−0.013	0.043	−0.016	0.759	−0.097	0.071			
	Kantianism	−0.051	0.037	−0.064	0.175	−0.124	0.023			
Internet-Interpersonal Conflicts	(Intercept)	2.137	0.285		<0.001	1.577	2.698	0.09	0.100 (>0.99)	5.510 (*p* < 0.001)
	Age	−0.013	0.004	−0.165	**<0.001**	−0.021	−0.006			
	Sex	−0.028	0.055	−0.025	0.612	−0.135	0.08			
	Narcissism	−0.041	0.043	−0.044	0.352	−0.126	0.045			
	Machiavellianism	0.073	0.045	0.094	0.106	−0.016	0.161			
	Psychopathy	0.104	0.044	0.14	**0.019**	0.017	0.19			
	Faith in humanity	0.048	0.034	0.072	0.162	−0.019	0.116			
	Humanism	−0.058	0.042	−0.074	0.164	−0.14	0.024			
	Kantianism	−0.021	0.036	−0.028	0.564	−0.092	0.05			
Mobile Phone-Conflicts	(Intercept)	2.372	0.303		<0.001	1.778	2.967	0.14	0.158 (>0.99)	8.740 (*p* < 0.001)
	Age	−0.016	0.004	−0.184	**<0.001**	−0.024	−0.008			
	Sex	0.026	0.058	0.022	0.648	−0.087	0.14			
	Narcissism	−0.142	0.046	−0.143	**0.002**	−0.233	−0.051			
	Machiavellianism	0.146	0.048	0.174	**0.002**	0.053	0.24			
	Psychopathy	0.082	0.047	0.102	0.08	−0.01	0.174			
	Faith in humanity	0.029	0.036	0.04	0.419	−0.042	0.101			
	Humanism	−0.026	0.044	−0.031	0.553	−0.113	0.061			
	Kantianism	−0.11	0.038	−0.137	**0.004**	−0.186	−0.035			
Mobile Phone-Communicational Emotional Usage	(Intercept)	1.665	0.275		<0.001	1.125	2.206	0.14	0.157 (>0.99)	8.674 (*p* < 0.001)
	Age	−0.012	0.004	−0.151	**<0.001**	−0.019	−0.005			
	Sex	0.135	0.053	0.124	**0.011**	0.031	0.238			
	Narcissism	−0.001	0.042	−0.001	0.994	−0.083	0.082			
	Machiavellianism	0.213	0.043	0.28	**<0.001**	0.128	0.298			
	Psychopathy	0.054	0.042	0.073	0.206	−0.03	0.137			
	Faith in humanity	−0.015	0.033	−0.023	0.648	−0.08	0.05			
	Humanism	0.041	0.04	0.053	0.304	−0.038	0.12			
	Kantianism	−0.023	0.035	−0.031	0.512	−0.091	0.046			

Overall, individual model *F*-tests were significant for all five outcomes, even after Bonferroni correction (0.05/5 = 0.01). Individual *p*-values for predictors were not corrected. Significant *p*-values are presented in bold letters. Male participants were coded as “0” and females as “1.” There is no evidence for multicollinearity, as assessed through the Variance Inflation Factor (VIF): sex (1.209), age (1.049), Narcissism (1.100), Machiavellianism (1.645), Psychopathy (1.714), Faith in Humanity (1.275), Humanism (1.377), and Kantianism (1.158). Significant *p*-values are presented in bold.

A Multigroup Path Analysis was conducted to examine sex differences in predictors of digital behaviors and conflicts (see Table 3). Among females, Internet Gaming Disorder scores were positively predicted by Machiavellianism (*β* = 0.161, *p* = 0.022) and Psychopathy (*β* = 0.284, *p* < 0.001), with an overall model fit of *R*^2^ = 0.164, *p* < 0.001. Among males, Internet Gaming Disorder was similarly positively predicted by Machiavellianism (*β* = 0.178, *p* = 0.026) and Psychopathy (*β* = 0.223, *p* = 0.006), with a slightly higher overall model fit (*R*^2^ = 0.203, *p* < 0.001). These results suggest that while Machiavellianism and Psychopathy influence Internet Gaming Disorder across sexes, the effects are somewhat stronger in males.

**Table 3:** Multigroup path analysis based on respondent’s sex.

Group	Outcome	Predictor	Estimate	SE	95% *CI LL*	95% *CI UL*	*β*	*p*
Female	Internet Gaming Disorder	Age	−0.008	0.005	−0.019	0.003	−0.086	0.158
		Narcissism	−0.112	0.067	−0.244	0.02	−0.1	0.096
		Machiavellianism	0.16	0.07	0.023	0.297	0.161	**0.022**
		Psychopathy	0.288	0.069	0.153	0.424	0.284	**<0.001**
		Faith in humanity	0.095	0.052	−0.006	0.196	0.121	0.064
		Humanism	0.031	0.067	−0.101	0.163	0.032	0.645
		Kantianism	0.017	0.056	−0.093	0.127	0.019	0.763
	Internet-Intrapersonal Conflicts	Age	−0.004	0.005	−0.015	0.006	−0.05	0.431
		Narcissism	−0.056	0.065	−0.184	0.072	−0.053	0.388
		Machiavellianism	0.206	0.068	0.073	0.339	0.22	**0.002**
		Psychopathy	0.085	0.067	−0.047	0.216	0.089	0.206
		Faith in humanity	0.078	0.05	−0.02	0.176	0.105	0.12
		Humanism	−0.034	0.065	−0.162	0.094	−0.037	0.606
		Kantianism	−0.114	0.054	−0.22	−0.007	−0.134	**0.036**
	Internet-Interpersonal Conflicts	Age	−0.014	0.005	−0.024	−0.005	−0.185	**0.003**
		Narcissism	0.034	0.059	−0.082	0.15	0.036	0.568
		Machiavellianism	−0.012	0.062	−0.133	0.109	−0.014	0.844
		Psychopathy	0.165	0.061	0.046	0.285	0.192	**0.007**
		Faith in humanity	0.078	0.045	−0.011	0.167	0.116	0.086
		Humanism	−0.065	0.059	−0.181	0.051	−0.079	0.271
		Kantianism	−0.048	0.049	−0.144	0.049	−0.062	0.334
	Mobile phone-Conflicts	Age	−0.012	0.005	−0.022	−0.002	−0.151	**0.014**
		Narcissism	−0.108	0.06	−0.226	0.01	−0.108	0.074
		Machiavellianism	0.173	0.063	0.05	0.296	0.196	**0.006**
		Psychopathy	0.136	0.062	0.015	0.258	0.151	**0.028**
		Faith in humanity	0.044	0.046	−0.047	0.134	0.062	0.343
		Humanism	−0.003	0.06	−0.122	0.115	−0.004	0.954
		Kantianism	−0.085	0.05	−0.184	0.013	−0.106	0.089
	Mobile Phone-Communicational Emotional Usage	Age	−0.016	0.005	−0.026	−0.007	−0.199	**0.001**
		Narcissism	−0.056	0.062	−0.177	0.065	−0.054	0.367
		Machiavellianism	0.197	0.064	0.07	0.323	0.215	**0.002**
		Psychopathy	0.118	0.064	−0.007	0.242	0.126	0.064
		Faith in humanity	−0.02	0.047	−0.113	0.073	−0.027	0.678
		Humanism	0.015	0.062	−0.107	0.136	0.016	0.812
		Kantianism	−0.006	0.051	−0.107	0.095	−0.007	0.91
Male	Internet Gaming Disorder	Age	−0.018	0.01	−0.037	0.001	−0.123	0.055
		Narcissism	0.064	0.096	−0.124	0.251	0.045	0.506
		Machiavellianism	0.218	0.098	0.026	0.411	0.178	**0.026**
		Psychopathy	0.259	0.095	0.073	0.445	0.223	**0.006**
		Faith in humanity	−0.046	0.077	−0.197	0.105	−0.044	0.548
		Humanism	−0.045	0.086	−0.214	0.124	−0.037	0.6
		Kantianism	−0.082	0.079	−0.237	0.072	−0.07	0.297
	Internet-Intrapersonal Conflicts	Age	−0.02	0.006	−0.032	−0.008	−0.212	**<0.001**
		Narcissism	−0.105	0.06	−0.223	0.013	−0.117	0.082
		Machiavellianism	0.347	0.062	0.225	0.469	0.441	**<0.001**
		Psychopathy	0.017	0.06	−0.101	0.134	0.022	0.783
		Faith in humanity	0.042	0.049	−0.053	0.137	0.062	0.386
		Humanism	0.01	0.054	−0.097	0.116	0.012	0.859
		Kantianism	0.012	0.05	−0.086	0.109	0.016	0.812
	Internet-Interpersonal Conflicts	Age	−0.016	0.006	−0.028	−0.003	−0.169	**0.014**
		Narcissism	−0.085	0.064	−0.21	0.041	−0.096	0.186
		Machiavellianism	0.148	0.066	0.019	0.277	0.193	**0.025**
		Psychopathy	0.048	0.064	−0.077	0.172	0.065	0.453
		Faith in humanity	0.023	0.052	−0.078	0.124	0.035	0.654
		Humanism	−0.046	0.058	−0.159	0.067	−0.061	0.427
		Kantianism	0.004	0.053	−0.1	0.107	0.005	0.943
	Mobile phone-Conflicts	Age	−0.024	0.007	−0.037	−0.01	−0.223	**<0.001**
		Narcissism	−0.178	0.071	−0.316	−0.04	−0.179	**0.012**
		Machiavellianism	0.126	0.073	−0.016	0.269	0.145	0.082
		Psychopathy	0.039	0.07	−0.098	0.177	0.048	0.574
		Faith in humanity	0.013	0.057	−0.099	0.124	0.017	0.825
		Humanism	−0.05	0.064	−0.175	0.075	−0.058	0.433
		Kantianism	−0.144	0.058	−0.258	−0.03	−0.172	**0.013**
	Mobile Phone-Communicational Emotional Usage	Age	−0.007	0.006	−0.018	0.004	−0.079	0.239
		Narcissism	0.063	0.056	−0.047	0.172	0.08	0.261
		Machiavellianism	0.228	0.057	0.116	0.341	0.332	**<0.001**
		Psychopathy	−0.003	0.055	−0.111	0.106	−0.004	0.96
		Faith in humanity	−0.019	0.045	−0.107	0.069	−0.032	0.674
		Humanism	0.068	0.05	−0.031	0.166	0.1	0.18
		Kantianism	−0.022	0.046	−0.112	0.069	−0.033	0.638

The model achieved the following fit indices: *χ*^2^(90) = 1,075, *p* < 0.001, CFI > 0.99, TLI > 0.99, GFI > 0.99, RMSEA < 0.001. Significant *p*-values are presented in bold.

For Internet-related Intrapersonal Conflicts, females showed positive associations with Machiavellianism (*β* = 0.22, *p* = 0.002) and negative associations with Kantianism (*β* = −0.134, *p* = 0.036), with an overall model fit of *R*^2^ = 0.110, *p* < 0.001. Among males, Internet-related Intrapersonal Conflicts were positively predicted by Machiavellianism (*β* = 0.441, *p* < 0.001) and negatively predicted by age (*β* = −0.212, *p* < 0.001), with a notably higher overall model fit (*R*^2^ = 0.227, *p* < 0.001). These findings indicate that while Machiavellianism is a strong predictor across both sexes, its influence is markedly greater in males, whereas Kantianism plays a significant protective role only in females.

For Internet-related Interpersonal Conflicts, females exhibited positive predictions by Psychopathy (*β* = 0.192, *p* = 0.007) and negative predictions by age (*β* = −0.185, *p* = 0.003), with an overall model fit of *R*^2^ = 0.098, *p* < 0.001. In males, Machiavellianism was positively associated (*β* = 0.193, *p* = 0.025), and age was negatively associated (*β* = −0.169, *p* = 0.014), with an overall model fit of *R*^2^ = 0.090, *p* = 0.006. These results suggest that Psychopathy is a more salient factor for females, while Machiavellianism plays a more prominent role in males.

For Mobile Phone-related Conflicts, females showed negative predictions by age (*β* = −0.151, *p* = 0.014) and positive predictions by Machiavellianism (*β* = 0.196, *p* = 0.006) and Psychopathy (*β* = 0.151, *p* = 0.028), with an overall model fit of *R*^2^ = 0.150, *p* < 0.001. In contrast, males demonstrated negative predictions by age (*β* = −0.223, *p* < 0.001), Narcissism (*β* = −0.179, *p* = 0.012), and Kantianism (*β* = −0.172, *p* = 0.013), with an overall model fit of *R*^2^ = 0.139, *p* < 0.001. These differences highlight that Psychopathy is more influential in females, while Narcissism and Kantianism are more relevant in males.

Finally, for Mobile Phone-Communicational Emotional Usage, females showed positive predictions by Machiavellianism (*β* = 0.215, *p* = 0.002) and negative predictions by age (*β* = −0.199, *p* = 0.001), with an overall model fit of *R*^2^ = 0.159, *p* < 0.001. Among males, Machiavellianism was the sole significant predictor (*β* = 0.332, *p* < 0.001), with an overall model fit of *R*^2^ = 0.135, *p* < 0.001. This indicates that while Machiavellianism is consistently associated with emotional phone usage across sexes, age-related declines are observed only in females.

Overall, the analysis reveals both similarities and notable differences between the sexes. While Machiavellianism consistently emerges as a key predictor across both groups, its effects tend to be stronger in males. In contrast, traits such as Psychopathy, Kantianism, and Narcissism exhibit more sex-specific associations, influencing females and males differently across digital behaviors and conflicts.

## 4 Discussion

This study advances our understanding of the interplay between personality traits and digital behaviors by examining the Dark and Light Triad traits as predictors of Internet Gaming Disorder and intrapersonal and interpersonal conflicts related to Internet and mobile phone use. Furthermore, it reveals critical sex differences in these relationships, underscoring the importance of tailoring interventions to individual psychological profiles. These findings contribute significantly to the growing discourse on digital behavior, offering insights that bridge personality psychology and technology-mediated behaviors.

Our findings underscore the profound yet contrasting roles of Dark and Light Triad traits in influencing digital behaviors. Among the Dark Triad traits, Machiavellianism and Psychopathy emerged as dominant predictors of maladaptive behaviors, including IGD and digital conflicts. The manipulative and self-serving nature of Machiavellianism, combined with the impulsivity and emotional detachment characteristic of Psychopathy, may exacerbate excessive engagement with digital platforms, where such traits thrive in the absence of direct interpersonal accountability. These results align with prior evidence that individuals with these traits leverage the online environment to exploit, dominate, or escape real-world constraints, thereby increasing susceptibility to maladaptive outcomes (52, 53).

The current study’s findings align with prior research, indicating that Machiavellianism and Psychopathy are significant predictors of IGD (54), mainly through mechanisms such as psychological need satisfaction and negative coping styles. Our results similarly highlight the strong influence of Machiavellianism and Psychopathy on IGD, suggesting that individuals with these traits may engage in compensatory gaming behaviors to fulfill unmet psychological needs or as a maladaptive coping strategy. Interestingly, prior research has also demonstrated that Narcissism predicts IGD indirectly through negative coping styles, which provides an important context for interpreting the protective role of Narcissism observed in our study (55). While Narcissism may reduce intrapersonal conflicts in digital contexts, it is possible that, under specific conditions involving stress or maladaptive coping, this trait may still contribute to problematic gaming behaviors.

Additionally, most video games feature reward systems that act as powerful stimuli for individuals with Machiavellian and Psychopathic traits. Since those with high Dark Triad scores are often highly reward-oriented (56, 57), these systems may reinforce their engagement by appealing to their desire for dominance, competition, and immediate gratification. This dynamic may contribute to a cycle of increased gaming to satisfy these psychological drives, further exacerbating problematic gaming behaviors.

Similar patterns were observed with problematic internet use. Individuals who experienced interpersonal conflicts due to problematic internet use had high Machiavellianism scores. Conversely, higher Narcissism scores were associated with reduced intrapersonal conflict related to problematic internet use. Several studies, like the one made by Kircaburun and Griffiths (58), have found that problematic internet use is related to high levels of Machiavellianism. This is probably caused by people having unlimited access to activities and websites (video games, online shopping, social media) that encourage them to be online constantly. Additionally, excessive internet use may expose people to overwhelming online information and stimuli. This can lead to intrapersonal conflicts like decision-making difficulties, low self-esteem, and depression (59). On the other side, people with high psychopath levels showed more interpersonal conflicts related to the excessive use of the internet. This can be caused because people with psychopath traits can have difficulty establishing social relationships (60).

Our findings indicate that higher scores in conflictive mobile phone use were related to higher Machiavellianism traits. Conversely, Narcissism exhibited a surprising protective role, particularly in reducing intrapersonal and mobile phone-related conflicts. Although people with predominant Narcissism traits tend to spend more time using their mobile phones (61), previous studies have also reported an inverse relationship between Narcissism and the problematic behavior of mobile phones (62).

One plausible explanation is that narcissistic individuals, driven by a heightened desire to maintain their self-image and social status, may regulate their digital behaviors to avoid scenarios that could harm their public persona or create internal dissonance. An alternative explanation for this protective role is rooted in the social and self-enhancement strategies employed by narcissists. Their tendency to curate idealized digital personas and preference for controlled social interactions may result in fewer opportunities for digital conflicts. For instance, narcissistic individuals might strategically avoid contentious online interactions or limit their exposure to scenarios where they could lose face. An example is the tendency of individuals high in grandiosity, a subcomponent of Narcissism, to prefer private social media accounts over public ones (63).

Our study also suggests that individuals with high scores in Kantianism exhibit fewer conflicts related to mobile phone use. This trait, which emphasizes viewing others as ends in themselves rather than as means to an end, appears to act as a protective factor against the development of mobile phone dependency. Due to their ethical orientation, it can be inferred that such individuals are less likely to engage in mobile phone use for immediate gratification or conflict resolution, potentially fostering better emotional regulation and reducing negative effects such as social isolation or overreliance on technology. This perspective is supported by Aylsworth and Castro (64), who argue that the moral duty is to regulate our use of smartphones and other devices to preserve personal autonomy and well-being.

Additionally, our findings suggest that increases in age are associated with lower scores of problematic behaviors; this tendency was present in all the studied variables. This indicates that young adults are particularly vulnerable to these behaviors; public policies on mental health should prioritize this age group (65).

The multigroup path analysis revealed compelling sex differences that deepen our understanding of how personality traits differentially shape digital behaviors. Previous studies have suggested that women are more likely to use mobile phones to interact with others (66). For both sexes, Machiavellianism and Psychopathy significantly predicted IGD, yet the effects were stronger in males, suggesting that men’s gaming behaviors may be more tightly intertwined with opportunistic and impulsive tendencies. This heightened susceptibility in males may stem from sociocultural norms reinforcing risk-taking and dominance (67, 68), particularly in competitive gaming environments (69). Additionally, female players may be especially susceptible to harassment from male players, compounded by the prevalence of violence and overt sexual content often featured in video games (70–72).

In the context of intrapersonal conflicts related to internet use, Machiavellianism was a strong predictor across the sexes, yet its influence was markedly greater in males. This suggests that males with manipulative tendencies may struggle more intensely with the internal consequences of their online behaviors, such as feelings of guilt or cognitive dissonance (73, 74). Among females, Kantianism played a significant protective role, reinforcing that women with a strong respect for others may approach their online interactions with greater mindfulness and restraint (75, 76).

Interpersonal conflicts revealed a distinct pattern: Psychopathy emerged as a stronger predictor among females, whereas Machiavellianism was more influential for males. This difference suggests that women high in Psychopathy may engage in more emotionally volatile or impulsive digital interactions (77), whereas men high in Machiavellianism may adopt calculated strategies that strain their relationships (78, 79). These findings highlight the need for sex-specific interventions that address the unique pathways through which personality traits manifest in digital conflicts.

Notable differences were observed in mobile phone-related conflicts. Among females, both Psychopathy and Machiavellianism were significant predictors, reflecting a dual pathway of impulsivity and manipulation contributing to conflict (79, 80). Among males, narcissism and Kantianism displayed protective effects, suggesting that self-assuredness and principled respect for others may mitigate conflict in this group. The interaction between these traits can act as a protective mechanism in interpersonal conflicts, as narcissism provides self-confidence and emotional resilience, while Kantianism promotes ethical respect for others, fostering a more balanced and less destructive approach to conflict resolution (81, 82). This divergence emphasizes the importance of considering sex and personality interactions when designing strategies to address mobile phone-related issues.

Finally, communicational emotional usage was consistently predicted by Machiavellianism across the sexes, indicating that individuals high in this trait may leverage mobile communication for strategic or manipulative purposes (78, 83). However, females exhibited an additional age-related decline in emotional dependency on mobile communication, a trend not observed in males (84, 85). This finding may reflect broader developmental or sociocultural differences in how emotional connections are maintained across the lifespan.

The findings of this study offer important theoretical contributions to understanding how personality traits shape digital behaviors. By examining the Dark and Light Triad traits, the study highlights the need for an integrative framework that captures both maladaptive and adaptive dimensions of personality in the digital context. The protective role of Narcissism in reducing intrapersonal and mobile phone-related conflicts challenges traditional views of this trait as inherently detrimental, suggesting its influence is highly context-dependent and tied to self-regulation strategies. Additionally, identifying Light Triad traits, particularly Kantianism, as protective factors underscores their critical role in fostering prosocial, harmonious, and mindful digital behaviors. The observed sex differences further illuminate how sociocultural and contextual factors interact with personality traits, emphasizing the importance of considering gendered dynamics in future research.

The findings are consistent with the I-PACE model, which explores how personality traits, emotional states, and cognitive processes interact to shape problematic behaviors (86). Specifically, the influence of Dark and Light traits underscores the role of individual personality factors as predispositional variables. The I-PACE model also provides insight into how cognitive and affective processes interact with personality traits. Furthermore, the model’s flexibility allows for consideration of gender differences, as personality traits influence digital behaviors in distinct ways across men and women, highlighting the complexity of pathways to digital addiction.

The potential public health implications of these findings are meaningful. As digital technologies become increasingly integrated into daily life, understanding the role of personality traits in fostering problematic behaviors is crucial for developing effective prevention and intervention strategies. This study highlights the need for targeted mental health initiatives considering individual differences in susceptibility to technology-related problematic behaviors. Healthcare providers and policymakers can design more tailored approaches to mitigate the adverse effects of excessive technology use by identifying those at higher risk-particularly individuals exhibiting Dark Triad traits. Furthermore, the study underscores the importance of public health campaigns aimed at educating the public about the psychological risks associated with digital consumption, emphasizing the necessity of balanced and mindful technology use to protect mental well-being on a broader scale (87).

These results underscore the need for tailored interventions to promote digital well-being. For individuals high in Machiavellianism or Psychopathy, interventions that focus on emotional regulation, empathy-building, and ethical decision-making may mitigate their vulnerability to problematic digital behaviors. Conversely, fostering Light Triad traits through mindfulness training, empathy exercises, or values-based interventions may enhance resilience and reduce conflicts, particularly among women.

Despite the relevance of our findings, some limitations must be considered. First, the limited sample size and non-probabilistic participant selection may limit our results’ generalizability. Second, our study was based on self-reported measurements, which participants might have been biased in responding to. Third, the low internal consistency of the Kantianism subscale (*α* = 0.67) should be noted as a relevant limitation, as it suggests that the items may not be reliably measuring the intended construct. This could affect the validity of the results and calls for further refinement of the subscale in future studies, potentially by revising or adding items to enhance its reliability and better capture the nuances of Kantianism. Fourth, the cross-sectional design precludes causal inferences, and future longitudinal studies are needed to elucidate the temporal dynamics of these relationships.

Further research is needed to build on the evidence presented in our study and address key gaps in the understanding of behavioral addictions. Specifically, future studies should investigate the prevalence and predictors of behavioral addictions in low- and middle-income countries, as well as the development and effectiveness of corresponding psychosocial interventions. Additionally, examining the impact of behavioral addictions on mental health indicators, such as depression, anxiety, somatization, and overall well-being, would provide critical insights into their broader psychological consequences. Research should also explore the moderating influence of cultural and contextual factors, as sociocultural norms may significantly shape how personality traits manifest and affect digital behaviors. Moreover, experimental studies aimed at testing interventions that foster Light Triad traits or reduce the influence of Dark Triad tendencies could offer valuable strategies for promoting healthier digital habits and mitigating the negative outcomes associated with maladaptive personality traits.

In conclusion, this study offers a comprehensive and nuanced perspective on the intersection of personality traits and digital behaviors, revealing both universal patterns and critical sex differences. By highlighting the dual role of Dark and Light Triad traits, these findings underscore the complexity of personality-driven digital behaviors and point to promising avenues for intervention. As digital technologies continue to reshape human interactions, understanding the psychological underpinnings of these behaviors is essential for fostering digital well-being in an increasingly connected world.

## Data Availability

The original contributions presented in the study are included in the article/supplementary material, further inquiries can be directed to the corresponding author.

## References

[1] Lasko EN Chester DS. What makes a “successful” psychopath? Longitudinal trajectories of offenders' antisocial behavior and impulse control as a function of psychopathy. Personal Disord. (2021) 12:207–15. doi: 10.1037/per0000421, PMID: 32584094 PMC7759585

[2] Molinuevo Alonso B Garreta Muniello D Torrubia Beltri R Martínez Membrives E Bonillo Martín A Requena Martínez A. La tètrada fosca i la predicció de la conducta agressiva, antisocial i d’adaptació institucional en joves internats en centres educatius. Invesbreu Criminologia. (2018) 77:4–5.

[3] Boccio CM Beaver KM. Psychopathic personality traits and the successful criminal. Int J Offender Ther Comp Criminol. (2018) 62:4834–53. doi: 10.1177/0306624X18787304, PMID: 30066592

[4] Paulhus D Williams K. The dark triad of personality: narcissism, Machiavellianism, and psychopathy. J Res Pers. (2002) 36:556–63. doi: 10.1016/S0092-6566(02)00505-6

[5] González J Garita-Campos D Godoy-Izquierdo D. La triada oscura de la personalidad y sus implicaciones psicológicas en el deporte. Una revisión sistemática. Rev Cuadernos Psicol Deporte. (2018) 2:191–207.

[6] Kowalski CM Vernon PA Schermer JA. The dark triad and facets of personality. Curr Psychol. (2021) 40:5547–58. doi: 10.1007/s12144-019-00518-0

[7] Persson B. N. (2019). The latent structure of the dark triad: Unifying Machiavellianism and psychopathy. Doctoral thesis, University of Turku, Finland, UA.

[8] Paulhus D. Toward a taxonomy of dark personalities. Curr Dir Psychol Sci. (2014) 23:421–6. doi: 10.1177/0963721414547737

[9] Lukić P Živanović M. Shedding light on the light triad: further evidence on structural, construct, and predictive validity of the light triad. Personal Individ Differ. (2021) 178:110876. doi: 10.1016/j.paid.2021.110876

[10] Maralov VG. The light triad of personality: a review of foreign studies. J Mod Foreign Psychol. (2024) 13:18–30. doi: 10.17759/jmfp.2024130302

[11] Ramos-Vera C García O'Diana A Sánchez Villena A Bonfá-Araujo B de Oliveira Barros L Porto Noronha AP. Dark and light triad: a cross-cultural comparison of network analysis in 5 countries. Personal Individ Differ. (2023) 215:112377. doi: 10.1016/j.paid.2023.112377

[12] Curtis GJ. It Kant be all bad: contributions of light and dark triad traits to academic misconduct. Personal Individ Differ. (2023) 212:112262. doi: 10.1016/j.paid.2023.112262

[13] Mewara A. Effect of light triad, meaning in life on level of life satisfaction among health care workers: a comparative study. Int J Res Anal Rev. (2024) 11:73.

[14] Kaufman SB Yaden DB Hyde E Tsukayama E. The light vs. dark triad of personality: contrasting two very different profiles of human nature. Front Psychol. (2019) 10:467. doi: 10.3389/fpsyg.2019.00467, PMID: 30914993 PMC6423069

[15] Derevensky J Hayman V Lynette G. Behavioral addictions: excessive gambling, gaming, internet, and smartphone use among children and adolescents. Pediatr Clin N Am. (2019) 66:1163–82. doi: 10.1016/j.pcl.2019.08.008, PMID: 31679605

[16] Petry N Zajac K Ginley M. Behavioral addictions as mental disorders: to be or not to be? Annu Rev Clin Psychol. (2018) 14:399–423. doi: 10.1146/annurev-clinpsy-032816-045120, PMID: 29734827 PMC5992581

[17] Nikbin D Taghizadeh SK Rahman SA. Linking dark triad traits to Instagram addiction: the mediating role of motives. Technol Soc. (2022) 68:101892. doi: 10.1016/j.techsoc.2022.101892

[18] Castagna PJ Hart W. Light triad traits moderate the relationship between the dark tetrad and immoral character. Personal Individ Differ. (2024) 222:112593. doi: 10.1016/j.paid.2024.112593

[19] Caplan SE. Theory and measurement of generalized problematic internet use: a two-step approach. Comput Hum Behav. (2010) 26:1089–97. doi: 10.1016/j.chb.2010.03.012

[20] Kuss DJ Griffiths MD Karila L Billieux J. Internet addiction: A systematic review of epidemiological research for the last decade. Curr. Pharm. Des. (2014) 20:4026–4052. doi: 10.2174/13816128113199990617 24001297

[21] Cano García M Bustamante Agudelo D Espinosa Osorio LP Gaviria Cano V Gil Jaramillo LF González González V. Redes sociales: atrapamiento o conexión emocional. 1st ed. Medellín, Colombia: Editorial SEDUNAC, Corporación Universitaria Adventista. (2023).

[22] Moral V De La M Suárez C. Factores de riesgo en el uso problemático de Internet y del teléfono móvil en adolescentes españoles. Rev Iberoam Psicol Salud. (2016) 7:69–78. doi: 10.1016/j.rips.2016.03.001

[23] Vondráčková P Gabrhelík R. Prevention of internet addiction: a systematic review. J Behav Addict. (2016) 5:568–79. doi: 10.1556/2006.5.2016.085, PMID: 27998173 PMC5370363

[24] Weinstein A Lejoyeux M. Internet addiction or excessive internet use. Am J Drug Alcohol Abuse. (2010) 36:277–83. doi: 10.3109/00952990.2010.491880, PMID: 20545603

[25] Bernal-Ruiz C Rosa-Alcázar Á Rosa-Alcázar AI. Uso problemático de internet e impacto negativo de WhatsApp en universitarios españoles: Las emociones negativas como factor de riesgo. Behav Psychol. (2021) 29:297–311. doi: 10.51668/bp.8321205s

[26] Cheng Y Tseng P Lin P Chen T Stubbs B Carvalho A. Internet addiction and its relationship with suicidal behaviors: a meta-analysis of multinational observational studies. J Clin Psychiatry. (2018) 79:17r11761. doi: 10.4088/JCP.17r11761, PMID: 29877640

[27] Kawabe K Horiuchi F Ochi M Oka Y Ueno S. Internet addiction: prevalence and relation with mental states in adolescents. Psychiatry Clin Neurosci. (2016) 70:405–12. doi: 10.1111/pcn.12402, PMID: 27178110

[28] Casas JA Ruiz-Olivares R Ortega-Ruiz R. Validation of the internet and social networking experiences questionnaire in Spanish adolescents. Int J Clin Health Psychol. (2013) 13:40–8. doi: 10.1016/S1697-2600(13)70006-1

[29] Beranuy-Fargues M Chamarro-Lusar A Graner-Jordania C Carbonell-Sánchez X. Validación de dos escalas breves para evaluar la adicción a Internet y el abuso de móvil. Psicothema. (2009) 21:480–5. 19622333

[30] Bisen S Deshpande Y. Understanding internet addiction: a comprehensive review. Ment Health Rev J. (2018) 23:165–84. doi: 10.1108/MHRJ-07-2017-0023

[31] Morales Rodríguez FM Giménez Lozano JM Linares Mingorance P Pérez-Mármol JM. Influence of smartphone use on emotional, cognitive and educational dimensions in university students. Sustain For. (2020) 12:6646. doi: 10.3390/su12166646

[32] Lopez-Fernandez O Kuss D Romo L Morvan Y Kern L Graziani P. Self-reported dependence on mobile phones in young adults: a European cross-cultural empirical survey. J Behav Addict. (2017) 6:168–77. doi: 10.1556/2006.6.2017.020 28425777 PMC5520117

[33] Sahu M Gandhi S Sharma M. Mobile phone addiction among children and adolescents: a systematic review. J Addict Nurs. (2019) 30:261–8. doi: 10.1097/JAN.0000000000000309 31800517

[34] World Health Organization. (2015). Public health implications of excessive use of the internet, computers, smartphones and similar electronic devices: meeting report, main meeting hall, Foundation for Promotion of Cancer Research, National Cancer Research Centre, Tokyo, Japan. Retrieved December 2, 2020. Available at: https://apps.who.int/iris/handle/10665/184264

[35] Landa-Blanco M Herrera T Esponoza H Girón K Moncada S Cortés-Ramos A. The impact of benevolent childhood experiences on adult flourishing: the mediating role of light triad traits. Front Psychol. (2024) 15:1320169. doi: 10.3389/fpsyg.2024.1320169, PMID: 38721318 PMC11076749

[36] Landa-Blanco M Reyes-García Y Landa-Blanco AL Cortés-Ramos A Paz-Maldonado E. Social media addiction relationship with academic engagement in university students: the mediator role of self-esteem, depression, and anxiety. Heliyon. (2024) 10:e24384. doi: 10.1016/j.heliyon.2024.e24384, PMID: 38293527 PMC10825341

[37] Landa-Blanco M Romero K Caballero I Gálvez-Pineda E Fúnes-Henríquez MJ Romero R. Exploring suicide ideation in university students: sleep quality, social media, self-esteem, and barriers to seeking psychological help. Front Psych. (2024) 15:1352889. doi: 10.3389/fpsyt.2024.1352889, PMID: 38645419 PMC11027559

[38] World Health Organization. (2018). Addictive behaviours: gaming disorder. Retrieved December 3, 2020. Available at: https://www.who.int/news-room/q-a-detail/addictive-behaviours-gaming-disorder

[39] Mehroof M Griffiths MD. Online gaming addiction: the role of sensation seeking, self-control, neuroticism, aggression, state anxiety, and trait anxiety. Cyberpsychol Behav Soc Netw. (2010) 13:313–6. doi: 10.1089/cyber.2009.0229 20557251

[40] Gros L Debue N Lete J van de Leemput C. Video game addiction and emotional states: possible confusion between pleasure and happiness? Front Psychol. (2020) 10:2894. doi: 10.3389/fpsyg.2019.02894, PMID: 32047450 PMC6996247

[41] Feng W Ramo D Chan S Bourgeois J. Internet gaming disorder: Trends in prevalence 1998-2016. Addict Behav. (2017) 75:17–24. doi: 10.1016/j.addbeh.2017.06.010, PMID: 28662436 PMC5582011

[42] Wittek C Finserås T Pallesen S Mentzoni R Hanss D Griffiths M. Prevalence and predictors of video game addiction: a study based on a National Representative Sample of gamers. Int J Ment Heal Addict. (2016) 14:672–86. doi: 10.1007/s11469-015-9592-8, PMID: 27688739 PMC5023737

[43] Griffiths M Kuss D King D. Video game addiction: past, present and future. Curr Psychiatry Res Rev. (2012) 8:308–18. doi: 10.2174/157340012803520414

[44] Ferguson C. The good, the bad and the ugly: a meta-analytic review of positive and negative effects of violent video games. Psychiatry Q. (2017) 78:309–16. doi: 10.1007/s11126-007-9056-9, PMID: 17914672

[45] Granic I Lobel A Engels RC. The benefits of playing video games. Am Psychol. (2014) 69:66–78. doi: 10.1037/a0034857 24295515

[46] Primack B Carroll M McNamara M Klem M King B Rich M. Role of video games in improving health-related outcomes: a systematic review. Am J Prev Med. (2012) 42:630–8. doi: 10.1016/j.amepre.2012.02.023, PMID: 22608382 PMC3391574

[47] Brand M Wegmann E Stark R Müller A Wölfling K Robbins TW. The interaction of person-affect-cognition-execution (I-PACE) model for addictive behaviors: update, generalization to addictive behaviors beyond internet-use disorders, and specification of the process character of addictive behaviors. Neurosci Biobehav Rev. (2019) 104:1–10. doi: 10.1016/j.neubiorev.2019.06.032, PMID: 31247240

[48] Beranuy M Machimbarrena J Vega-Osés A Carbonell X Griffiths M Pontes H. Spanish validation of the internet gaming disorder scale-short form (IGDS9-SF): prevalence and relationship with online gambling and quality of life. Int J Environ Res Public Health. (2020) 17:1562. doi: 10.3390/ijerph17051562, PMID: 32121280 PMC7084394

[49] Jones D Paulhus D. Introducing the short dark triad (SD3): a brief measure of dark personality traits. Assessment. (2013) 21:28–41. doi: 10.1177/1073191113514105 24322012

[50] The jamovi project (2024). Jamovi (version 2.3.28) [computer software]. Available at: https://www.jamovi.org

[51] Cohen J. A power primer. Quant Methods Psychol. (1992) 112:155–9. doi: 10.1037//0033-2909.112.1.155, PMID: 19565683

[52] Csordas A Book A Worth N Visser B. The WoW factor: psychopathic traits and behavior in a massive multiplayer online role-playing game. Personal Individ Differ. (2022) 187:111443. doi: 10.1016/j.paid.2021.111443

[53] Hussain U Jabarkhail S Cunningham GB Madsen JA. The dual nature of escapism in video gaming: a meta-analytic approach. Comput Hum Behav Reports. (2021) 3:100081. doi: 10.1016/j.chbr.2021.100081

[54] Tang WY Reer F Quandt T. The interplay of gaming disorder, gaming motivations, and the dark triad. J Behav Addict. (2020) 9:491–6. doi: 10.1556/2006.2020.00013, PMID: 32544080 PMC8939412

[55] Xu X Gao LF Lian SL Chen Q Zhou ZK. How the dark triad is associated with internet gaming disorder? The serial mediation of basic psychological needs satisfaction and negative coping styles. Curr Psychol. (2022) 1-9:1–9. doi: 10.1007/s12144-022-03996-x, PMID: 36471813 PMC9714411

[56] Jonason PK Jackson CJ. The dark triad traits through the lens of reinforcement sensitivity theory. Personal Individ Differ. (2016) 90:273–7. doi: 10.1016/j.paid.2015.11.023

[57] Malesza M Kalinowski K. Dark triad and impulsivity—an ecological momentary assessment approach. Curr Psychol. (2021) 40:3682–90. doi: 10.1007/s12144-019-00320-y

[58] Kircaburun K Griffiths M. The dark side of internet: preliminary evidence for the associations of dark personality traits with specific online activities and problematic internet use. J Behav Addict. (2018) 7:993–1003. doi: 10.1556/2006.7.2018.109, PMID: 30427212 PMC6376394

[59] Anju A Amandeep A Punia BK Punia V Garg N. Life dissatisfaction among students: exploring the role of intrapersonal conflict, insufficient efforts and academic stress. Rajagiri Manag J. (2021) 15:113–28. doi: 10.1108/RAMJ-09-2020-0058

[60] Sindermann C Sariyska R Lachmann B Brand M Montag C. Associations between the dark triad of personality and unspecified/specific forms of internet-use disorder. J Behav Addict. (2018) 7:985–92. doi: 10.1556/2006.7.2018.114, PMID: 30541336 PMC6376366

[61] Gökçearslan S Yildiz DH Berikan B Saritepeci M. Smartphone addiction, loneliness, narcissistic personality, and family belonging among university students: a path analysis. Soc Sci Q. (2021) 102:1743–60. doi: 10.1111/ssqu.12949

[62] Hussain Z Griffiths MD Sheffield D. An investigation into problematic smartphone use: the role of narcissism, anxiety, and personality factors. J Behav Addict. (2017) 6:378–86. doi: 10.1556/2006.6.2017.052, PMID: 28849667 PMC5700726

[63] Nardis Y Panek E. Explaining privacy control on Instagram and twitter: the roles of narcissism and self-esteem. Commun Res Rep. (2018) 36:24–34. doi: 10.1080/08824096.2018.1555522

[64] Aylsworth T Castro C. Is there a duty to be a digital minimalist? J Appl Philos. (2021) 38:662–73. doi: 10.1111/japp.12498

[65] Lehtimaki S Martic J Wahl B Foster K Schwalbe N. Evidence on digital mental health interventions for adolescents and young people: systematic overview. JMIR Ment Health. (2021) 8:e25847. doi: 10.2196/25847, PMID: 33913817 PMC8120421

[66] De-Sola Gutiérrez J Rodríguez de Fonseca F Rubio G. Cell-phone addiction: a review. Front Psych. (2016) 7:1–15. doi: 10.3389/fpsyt.2016.00175, PMID: 27822187 PMC5076301

[67] Baker MD Maner JK. Risk-taking as a situationally sensitive male mating strategy. Evol Hum Behav. (2008) 29:391–5. doi: 10.1016/j.evolhumbehav.2008.06.001

[68] Brand JA Henry J Melo GC Wlodkowic D Wong BBM Martin JM. Sex differences in the predictability of risk-taking behavior. Behav Ecol. (2023) 34:108–16. doi: 10.1093/beheco/arac105, PMID: 36789395 PMC9918862

[69] Gisbert-Pérez J Martí-Vilar M Merino-Soto C Chans GM Badenes-Ribera L. Gender differences in internet gaming among university students: a discriminant analysis. Front Psychol. (2024) 15:1412739. doi: 10.3389/fpsyg.2024.1412739, PMID: 39569103 PMC11577638

[70] Lopez-Fernandez O Williams A Griffiths M Kuss D. Female gaming, gaming addiction, and the role of women within gaming culture: a narrative literature review. Front Psych. (2019) 10:1–14. doi: 10.3389/fpsyt.2019.00454, PMID: 31354536 PMC6635696

[71] McLean L Griffiths MD. Female gamers’ experience of online harassment and social support in online gaming: a qualitative study. Int J Ment Heal Addict. (2019) 17:970–94. doi: 10.1007/s11469-018-9962-0

[72] Skowronski M Busching R Krahé B. The effects of sexualized video game characters and character personalization on women's self-objectification and body satisfaction. J Exp Soc Psychol. (2021) 92:104051. doi: 10.1016/j.jesp.2020.104051

[73] Muñoz García A Gil-Gómez de Liaño B Pascual-Ezama D. Gender differences in individual dishonesty profiles. Front Psychol. (2021) 12:728115. doi: 10.3389/fpsyg.2021.728115, PMID: 34955957 PMC8703141

[74] Parent MC Gobble TD Rochlen A. Social media behavior, toxic masculinity, and depression. Psychol Men Masculinity. (2019) 20:277–87. doi: 10.1037/men0000156, PMID: 38250140 PMC10798810

[75] Glover SH Bumpus MA Sharp GF Munchus GA. Gender differences in ethical decision making. Women Manag Rev. (2002) 17:217–27. doi: 10.1108/09649420210433175

[76] Ochnik D Dembińska A. Gender differences and areas of internet behavior in seven years’ perspective. Pol Psychol Bull. (2018) 49:383–90. doi: 10.24425/119506

[77] Međedović J Wertag A Sokić K. Can psychopathic traits be adaptive? Sex differences in relations between psychopathy and emotional distress. Psihologijske Teme. (2018) 27:481–97. doi: 10.31820/pt.27.3.7

[78] Abell L Brewer G. Machiavellianism, self-monitoring, self-promotion and relational aggression on Facebook. Comput Hum Behav. (2014) 36:258–62. doi: 10.1016/j.chb.2014.03.076

[79] Collison KL South S Vize CE Miller JD Lynam DR. Exploring gender differences in Machiavellianism using a measurement invariance approach. J Pers Assess. (2020) 103:258–66. doi: 10.1080/00223891.2020.1729773, PMID: 32130029

[80] Hidalgo-Fuentes S. Uso problemático del smartphone: el papel de los Cinco Grandes, la Tríada Oscura y la impulsividad. Aloma Rev Psicol Ciències Educ Esport. (2021) 39:17–26. doi: 10.51698/aloma.2021.39.1.17-26

[81] Grijalva E Newman DA Tay L Donnellan MB Harms PD Robins RW. Gender differences in narcissism: a meta-analytic review. Psychol Bull. (2015) 141:261–310. doi: 10.1037/a0038231, PMID: 25546498

[82] Mathäs A. Mantener a raya el narcisismo: Kant y Schiller sobre lo sublime. Konturen. (2010) 3:19–44. doi: 10.5399/uo/konturen.3.1.1371

[83] Sheynov VP Yermak VO. Relationships between problematic smartphone use and Machiavellianism, personality orientation and communication skills. Pedagogical Rev. (2024) 1:115–22. doi: 10.23951/2307-6127-2024-1-115-122

[84] Hartung J Bader M Moshagen M Wilhelm O. Age and gender differences in socially aversive (“dark”) personality traits. Eur J Personal. (2022) 36:3–23. doi: 10.1177/0890207020988435

[85] Muscanell NL Guadagno RE. Make new friends or keep the old: gender and personality differences in social networking use. Comput Hum Behav. (2012) 28:107–12. doi: 10.1016/j.chb.2011.08.016

[86] Mehmood A Bu T Zhao E Zelenina V Nikishov A Wang W. Exploration of psychological mechanism of smartphone addiction among international students of China by selecting the framework of the I-PACE model. Front Psychol. (2021) 12:758610. doi: 10.3389/fpsyg.2021.758610, PMID: 34867657 PMC8632695

[87] Yosep I Suryani S Mediani HS Mardhiyah A Ibrahim K. Types of digital mindfulness: improving mental health among college students – a scoping review. J Multidiscip. (2024) 17:43–53. doi: 10.2147/JMDH.S443781, PMID: 38205126 PMC10777865

